# Early Retinal Function Deficit without Prominent Morphological Changes in the R6/2 Mouse Model of Huntington’s Disease

**DOI:** 10.1371/journal.pone.0113317

**Published:** 2014-12-03

**Authors:** Symantas Ragauskas, Henri Leinonen, Jooseppi Puranen, Seppo Rönkkö, Soile Nymark, Kestutis Gurevicius, Arto Lipponen, Outi Kontkanen, Jukka Puoliväli, Heikki Tanila, Giedrius Kalesnykas

**Affiliations:** 1 Department of Ophthalmology, Institute of Clinical Medicine, School of Medicine, University of Eastern Finland, Kuopio, Finland; 2 Vilnius Gediminas Technical University, Department of Biochemistry, Vilnius, Lithuania; 3 State Research Institute for Innovative Medicine, Vilnius, Lithuania; 4 Experimentica Ltd., Kuopio, Finland; 5 Department of Neurobiology, A.I. Virtanen Institute, University of Eastern Finland, Kuopio, Finland; 6 Department of Electronics and Communications Engineering, BioMediTech, Tampere University of Technology, Tampere, Finland; 7 Charles River DRS Finland, Kuopio, Finland; The University of Melbourne, Australia

## Abstract

Huntington’s disease (HD) is an inherited neurodegenerative disorder that primarily affects the medium-size GABAergic neurons of striatum. The R6/2 mouse line is one of the most widely used animal models of HD. Previously the hallmarks of HD-related pathology have been detected in photoreceptors and interneurons of R6/2 mouse retina. Here we aimed to explore the survival of retinal ganglion cells (RGCs) and functional integrity of distinct retinal cell populations in R6/2 mice. The pattern electroretinography (PERG) signal was lost at the age of 8 weeks in R6/2 mice in contrast to the situation in wild-type (WT) littermates. This defect may be attributable to a major reduction in photopic ERG responses in R6/2 mice which was more evident in b- than a-wave amplitudes. At the age of 4 weeks R6/2 mice had predominantly the soluble form of mutant huntingtin protein (mHtt) in the RGC layer cells, whereas the aggregated form of mHtt was found in the majority of those cells from the 12-week-old R6/2 mice and onwards. Retinal astrocytes did not contain mHtt deposits. The total numbers of RGC layer cells, retinal astrocytes as well as optic nerve axons did not differ between 18-week-old R6/2 mice and their WT controls. Our data indicate that mHtt deposition does not cause RGC degeneration or retinal astrocyte loss in R6/2 mice even at a late stage of HD-related pathology. However, due to functional deficits in the rod- and cone-pathways, the R6/2 mice suffer progressive deficits in visual capabilities starting as early as 4 weeks; at 8 weeks there is severe impairment. This should be taken into account in any behavioral testing conducted in R6/2 mice.

## Introduction

Huntington’s disease (HD) is an autosomal dominant neurodegenerative disorder which is caused by a CAG repeat expansion in the *Huntingtin* gene on chromosome 4 [1]–[2]. In normal individuals the number of CAG repeats varies between 16 and 20, whereas HD-affected individuals have a tract of 36 CAG repeats and more [3]. As a result, an expanded polyglutamine tract leads to the production of mutant form of huntingtin protein (mHtt). Abnormally produced mHtt is prone to aggregate. The exact mechanism of mHtt aggregation is still unknown, but a number of evidence suggests the existence of several mechanisms employing abnormally expanded repeats of glutamine (for a review see [4]). However, mHtt can co-exist in the cell as monomer, oligomer and large cytoplasmic and nuclear inclusions [5]–[7]. Amongst all conformational states, small oligomers of mHtt were proposed to be the most toxic form of mHtt [8].

The main and primary pathological hallmark of HD is loss of medium spiny GABAergic neurons in the striatum. As the disease progresses, neuronal loss is observed also in the neocortex and other subcortical regions. Clinically, HD-affected individuals suffer progressive motor dysfunctions with involuntary stereotypic movements and cognitive impairment during the later stages of the disease. Many well known neurodegenerative diseases, like Alzheimer's disease and Parkinson's disease, manifest also in the retina (for a review see [9]), but in HD retinal involvement is uncertain.

Developmentally, the retina and optic nerve are diencephalic extensions of the brain and are normally considered as a part of CNS. The activation of the retina can be non-invasively recorded by electroretinography (ERG) where the obtained waveform represents overlapping activation from distinct generators. However, specific signal collection and analysis techniques allow researchers to obtain rather cell specific signals from the retina (see [10] for a review). Moreover, characteristics such as well-defined and layered retinal anatomy, an immune privileged environment and transparency, which enables ocular imaging (fundoscopy and optical coherence tomography), means that the eye is a valuable model for studying neurodegenerative diseases.

There are few human studies on HD-related pathology in the eye and even the published papers are often contradictory. The involvement of the retina in the neurodegenerative process of HD has been reported previously based on performance in a visual test [11]. The researchers observed impairment in retinal increment thresholds in HD patients but not in patients with Tourette's syndrome or schizophrenia. This was evaluated in a behavioral assay which requires normal crosstalk between different cone subtypes. However, a case study of a 69-year-old male patient with a 28-year confirmed history of HD could find no signs of retinal pathology macroscopically, microscopically or at the ultrastructural level [12]. These results from human studies are in contradiction with previous findings on degenerative changes in retinas from HD mouse models [13]–[14]. Thus, there is a clear need to clarify whether HD pathology in animal models and in humans is manifested in the retina at an early stage of the disease.

A number of HD animal models are available; the R6/1 and R6/2 mouse lines have proved the most popular for studying HD-related pathogenesis and therapeutic applications. The R6/2 mice display the most severe HD-related neurological phenotype: deteriorations in performances in Morris water maze, visual cliff, two-choice swim tank, and T-maze tasks have been detected starting at 3.5 weeks of age [15]. However, uniform behavioral motor deficits appear as early as 5–6 weeks of age, become evident at about 9 weeks of age, and death in R6/2 mice usually occurs at an early age, 10–13 weeks [16]. Previous reports from R6/2 transgenic mice have indicated that mHtt expression levels in their retinas are comparable to that found in the brain [17]. Predominantly nuclear mHtt aggregates were found in all three nuclear layers of retina with large and numerous aggregates in the retinal ganglion cell layer (RGCL) neurons [17]. A recent study by Li et al. [14] showed progressive retinal pathology in R6/2 mice, such that cone-mediated ERG responses declined already at 6 weeks of age while a significant decrease in the cone nuclei count was observed starting from 10–11 weeks of age. The outer nuclear layer (ONL) had a wavy appearance in the progressed disease state which also displayed ONL disorganization. Another study which used the R6/1 line appeared to show identical retinal pathology only with a later onset and slower progression than R6/2 line, i.e. cone-specific dysregulation and functional deficit eventually leading to retinal remodeling, Müller cell gliosis and cell death [13].

Since all previously published studies have explored retinal pathology in R6/2 mice mainly at the photoreceptor and interneuron level, we have investigated in detail whether mHtt accumulation causes RGC dysfunction and degeneration in R6/2 mice. In addition, the presence of mHtt in retinal and optic nerve astrocytes and their survival in this animal model were studied. We also hypothesized that a functional alteration in the retina, especially in the cone-pathway, could precede overt motor symptoms; therefore we performed ERG measurements before the onset of motor symptoms at the mouse age of 4 weeks.

## Materials and Methods

### Animals

R6/2 transgenic mice carrying the N-terminal region of a mutant human *Huntingtin* gene [16] were used in this study. Mice were bred at Charles River Labs, Germany by crossing ovarian transplanted wild-type (WT) females carrying ovaries from R6/2 females (Jackson Laboratories, USA; stock no. 006494) with C57Bl/6J males (Charles River Labs, Germany). The R6/2 mice (n = 42) and their WT littermate controls (n = 40) were used in this study. The animals were individually housed at a constant temperature (22±1°C) and in a light-controlled environment (lights on from 7 am to 7 pm) with *ad libitum* access to food and water. All animals were treated in accordance with the ARVO Statement for the Use of Animals in Ophthalmic and Vision Research and the EC Directive 86/609/EEC for animal experiments, using protocols approved and monitored by the Animal Experiment Board of Finland.

### Electroretinography

Initially, 13 transgenic R6/2 and 7 wild-type mice were evaluated in ERG recordings. Ten R6/2 mice were measured at the age of 8 weeks and 6 at the age of 12 weeks. Only strong flashes (−1.50 to 0.50 log cd*s/m^2^) were used in ERG measurements at this age. Animals were dark-adapted overnight for at least 12 h prior to the recordings, and all preparations were performed under a dim red light. The animals were anesthetized with an intraperitoneal injection of ketamine (37.5 mg/kg; Ketalar, Pfizer Oy Animal Health, Helsinki, Finland) and medetomidine (0.45 mg/kg; Domitor, Orion Oy, Espoo, Finland) mixture. Subsequently, 0.3 ml of saline was injected subcutaneously in order to preserve cardiovascular homeostasis during the anesthesia. A drop of oxybuprocaine (Oftan-Obucain, Santen, Tampere, Finland) was applied on the cornea for local anesthesia and later replaced by thin layer of translucent eye lubricant (Lacri-Lube, Allergan, Upplands Väsby, Sweden) to prevent the corneas from drying during the experiment. The animal was placed on a controlled heating-pad (Biological Temperature Controller TMP-5b, Supertech Instruments, Pecs, Hungary) maintaining body temperature at approximately 37°C, and the nose was secured with a customized stereotaxic frame. To obtain representative PERG recordings, the eyes were not refracted for the viewing distance as the mouse eye has large depth of focus [18]–[19]. Under these conditions, the undilated mouse pupil points laterally and upwardly [20]–[21] and has a diameter less than 1 mm [22].

The recording electrode was a silver-wire (diameter 200 µm, PFA-insulated, A–M systems, Sequim, WA, USA) exposed and shaped as semicircular loop from the tip, inserted gently onto the posterior part of the right cornea by means of a micromanipulator. This simple electrode configuration was chosen to minimize the risk of cataract formation during anesthesia due to corneal irritation [20]–[21], [23]. Also, the electrode configuration does not interfere with vision and has been shown to generate highly reproducible results. A stainless steel injection needle (27G, Terumo Corporation, Tokyo, Japan) was inserted subcutaneously into the ipsilateral cheek from the stimulated eye to act as reference, and another electrode to the lower back in order to record the electrocardiogram, and a third on the tip of the tail which served as the common ground. The animal was further dark-adapted for 5 min without any light before initiation of scotopic flash stimuli. Because of the health concerns in R6/2 mice, we analyzed cardiac rhythm, breathing rate and QRS-complexes online and offline (data not shown). The anesthesia was immediately reversed by α2-antagonist for medetomidine, atipamezole (0,5 mg/kg i.p., Antisedan, Orion Oy, Espoo, Finland). The experimenter was initially blinded to the genotype before motor symptom onset.

### Stimulation

The flash ERG (FERG) stimuli were elicited with a single white LED using Grass S88 stimulator (Grass Medical Instruments, Quincy, MA, USA) connected with a stimulus isolator and current controller (Iso-Flex, A.M.P.I., Jerusalem, Israel) to obtain steady 5 ms flashes. The flash intensities were quantified with an optical power and energy meter (Thorlabs PM100D, Thorlabs, NJ, USA). To obtain clear rod-driven b-wave, −1.50 log cd*s/m^2^ intensity was used with a 4-s ISI, and averaged over 10–15 stimuli. This kind of stimulus elicits only a minimal a-wave and is thought to be purely mediated by rod-photoreceptors [24]. Scotopic 0.50 log cd*s/m^2^ flash with a 10-s ISI, averaged over 5–10 stimuli, was used to elicit primarily rod-driven but partially cone-affected mixed rod-cone response. Cone-responses were recorded after PERG recordings which served also as a light-adaptation method. Since PERG was not recorded at 12 weeks of age, the light-adaptation was done by intense constant LED-illumination for 5 min, followed by 1 min stabilization before initiation of 50–80 photopic flashes. The photopic 0.50 log cd*s/m^2^ flashes with a 1-s ISI were superimposed on a steady 5 cd/m^2^ rod-suppressing adapting field [25].

The PERG stimuli were displayed on a γ-linearized computer screen (21,5” LG IPS224T, LG Electronics, Seoul, South Korea) the center of which was aligned with the projection of the pupil from a distance of 19 cm, and the whole stimulus window covered 86.7 horizontal and 50.7 vertical degrees of visual field. The patterned stimuli consisted of vertical sinusoidal bars with reversing contrast at 1 Hz (2 reversals per second) generated by custom-made software in MATLAB (Mathworks, Natick, MA, USA), using the Psychophysics Toolbox extensions [26]–[27]. The patterned stimuli had a mean luminance of 60 cd/m^2^ and a 97% contrast between white and black gratings. The spatial frequency of the stimulus was 0.03 cycles per degree (CPD). In order to obtain the final PERG response, 200–300 stimuli for each image phase were first averaged, and then these averages were checked for consistency and finally superimposed.

### Data acquisition

Electrical signals generated in the retina were amplified (1000×) and band-pass filtered between 1–1000 Hz by an AC amplifier (gain 500, A–M Systems Inc., Sequim, WA, USA). The signals were digitized and recorded by using SciWorks 7.2 program (DataWave Technologies, Loveland, CO, USA). In addition, the signal from a customized light-sensor attached to the monitor, or an efferent copy signal from Grass stimulator, was acquired and used in the offline analysis for accurate event detection.

### ERG data analysis

**Figure 1:**
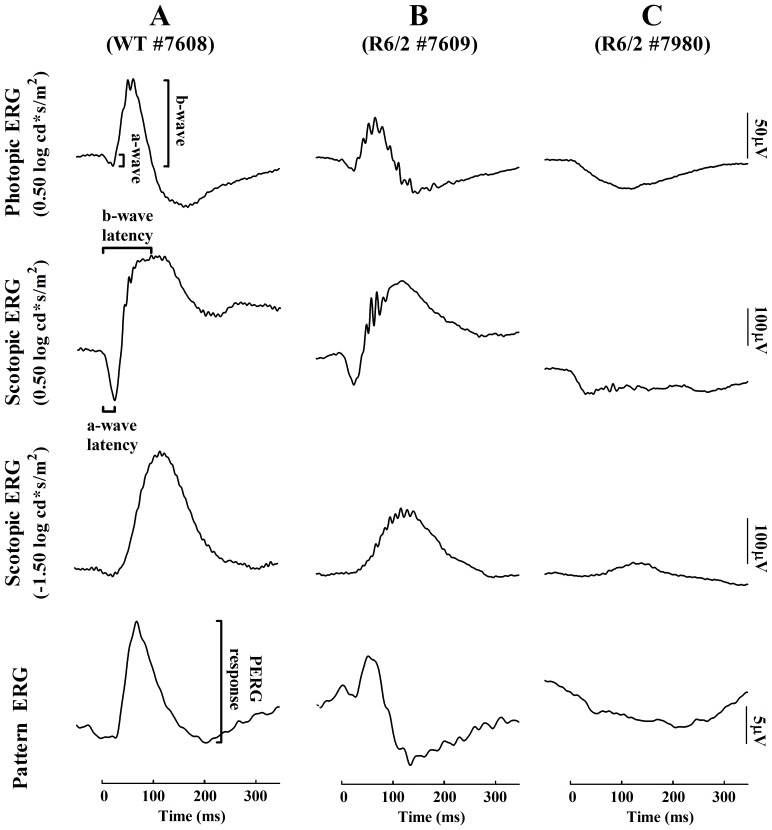
Representative ERG traces from one wild-type and two R6/2 mice for each recorded stimulus parameter at 8 weeks of age. Column A: Normal ERGs derived from a wild-type mouse. Column B: The least affected R6/2 mouse showing decreased amplitudes but preserved waveforms. Column C: A severely affected R6/2 mouse having only a hint of a minor positive going intrusion after a-wave for log 0.5 cd*s/m^2^ flashes, and a minor b-wave for the –log 1.5 cd*s/m^-2^ flash. Note: animals having no b-waves were cut out from further statistical analysis.

For each ERG response, a baseline was determined as the average amplitude at −100 to 0 ms from the stimulus onset. At higher luminous intensities, the photoreceptor elicited a-wave amplitude, and the latency was measured from the baseline to the first negative trough. The b-wave latency was measured from the first positive peak. The b-wave amplitude was measured from the a-wave trough for FERG and from the baseline for PERG. In addition, ratios between scotopic and photopic b- and a-waves, and PERG and photopic b-waves were calculated by division. The oscillatory potentials (OPs) were isolated by band-filtering (70–150 Hz) using a 2-way least-square FIR filter (i.e. the eegfilt.m routine from the EEGLAB toolbox) [28] and subtracted from the raw waveform to allow accurate a- and b-wave quantification. The OPs were not analyzed. R6/2 mice (n = 2−3 depending on the health condition and age) having only negative ERG (no b-waves) were excluded from statistical analysis in the 8-week and 12-week age groups. The analyzed ERG components are illustrated in Fig. 1A.

### Tissue preparation

The animals were deeply anesthetized with a mixture of ketamine (50 mg/kg; Ketalar, Pfizer Oy Animal Health) and medetomidine (0.4 mg/kg; Domitor, Orion Oy) injected intraperitoneally and perfused transcardially for 3 min with saline followed by 9 min with 4% paraformaldehyde in 0.1 M phosphate buffer solution (PB), pH 7.4. The eyes were enucleated and used for paraffin embedding or their retinas were detached from the sclera and post-fixed as wholemounts for 24 hours in the same fixative solution. Next, the retinas were washed with 0.1 M PB, pH 7.4, and processed for immunohistochemistry.

### Immunohistochemistry

All antibodies used in this study are listed in Table 1.

**Table 1:** Details of antibodies.

Antibody	Source (catalog number)	Dilution	Specificity
*Primary*			
Mouse anti-mHtt	Millipore (MAB5374)	1∶50 (paraffin sections) 1∶100 (retinal wholemounts)	Soluble and aggregated form of mutant Huntingtin protein
Mouse NeuN	Millipore (MAB377)	1∶100	Neuronal nuclei
Rabbit β3-tubulin	Cell Signaling Technology (D71G9)	1∶500	Cytoskeleton of neuronal cells. Retina: retinal ganglion cells
Rabbit anti-GFAP	DakoCytomation (Z0334)	1∶10 000	Macroglial cell marker
Rabbit anti-Iba1	Wako	1∶500	Microglial marker
*Secondary*			
*Goat anti-rabbit Alexa Fluor 488*	Life Technologies (A11008)	1∶500	Rabbit antibodies
*Goat anti-mouse Alexa Fluor 568*	Life Technologies (A11004)	1∶500	Mouse antibodies

### Wholemount retinas

The superior part of the eyes was identified for orientation, the eyes were enucleated, and the retinas were detached from the sclera and were post-fixed for 3 h in the same fixative solution. Subsequently, the retinas were washed in 0.1 M PB, pH 7.4, four times for 20 min, and 0.05 M Tris buffered saline (TBS), pH 7.4, two times for 20 min. Then the tissue was incubated in 10% normal goat serum (NGS; Colorado Serum Company, CO, USA) for 40 min, followed by washing in 1% NGS in TBS for 10 min and then incubated for 48 h at 4°C in mouse anti-mHtt (1∶100) primary antibody. This was followed by washing in 0.05 M TBS, pH 7.4, containing 1% NGS with 0.5% Triton, three times for 15 min, and incubation in goat anti-mouse Alexa Fluor 568 (1∶500) secondary antibody for 3 h at room temperature. Then the retinas were washed in 0.05 M TBS, pH 7.4, containing 1% NGS with 0.5% Triton, three times for 15 min, and incubated in rabbit anti-glial fibrillary acidic protein (GFAP; 1∶10 000) for 24 h at 4°C and goat anti-rabbit Alexa Fluor 488 (1∶500) for 3 h at room temperature. All antiserums were diluted in 0.05 M TBS, pH 7.4, containing 1% NGS (anti-mHtt, anti-GFAP, Alexa Fluor 568, Alexa Fluor 568). The sections were counterstained with 4′,6-diamidino-2-phenylindole (DAPI, 1∶10 000 in 0.05 M TBS, pH 7.4). Control stainings for immunohistochemistry were carried out by the omission of the primary antibodies. No evidence of any staining was observed in these negative controls. The stained sections were flat mounted on a glass slide in glycerol with the RGC layer on top and coverslipped.

### Stereology of retinal cells

The optical fractionator method [29] was used to estimate the total numbers of cells from wholemount retinas. Briefly, DAPI-positive cells from the RGCL and GFAP-immunoreactive (ir) profiles from the nerve fiber layer (NFL) were counted manually using the Stereo Investigator software (MicroBrightField, VT, USA). The fractionator sampling consisted of a section sampling fraction (ssf), an area sampling fraction (asf) and a height sampling fraction (hsf). The ssf for each retina was equal to 1, asf was 0.0016 (DAPI) and 0.01 (GFAP-positive profiles). The hsf was equal to 1. The retina was first outlined using CFI Plan Achro 4× objective (N.A. 0.1, W.D. 30). Thereafter, a CFI Plan Fluor 100× oil immersion objective (N.A. 1.30, W.D. 0.20) was used for DAPI counting. A CFI Plan Achro 40× (N.A. 0.95, W.D. 0.14) objective was used to count GFAP-positive profiles in the retina. The mean thickness of the mounted sections was 120 µm. The plane for the top of the counting frame in the z-dimension was set to 0 µm from the surface of the section. The cells were counted while focusing 120 µm through the section. The reference criterion for the counting was the cell body.

### Stereology of optic nerve axons

After perfusion fixation, the optic nerves were placed in 1% osmium, dehydrated in ascending alcohol concentrations, and then placed in 1% uranyl acetate in 100% ethanol for 1 hour. Subsequently the optic nerves were embedded in epoxy resin mixture at 60°C for 48 hours. One micron-thick cross-sections of the optic nerve were cut and stained using toluidine blue. The optical fractionator method [29] was used to estimate the total number of axons. The optic nerve section was first outlined using CFI Plan Fluor 100× oil immersion objective (N.A. 1.30, W.D. 0.20). The sampling step used for the stage movement was 70 µm for the x-axis and 70 µm for the y-axis. The counting frame of 10 µm×10 µm was placed randomly overdrawn contour by the motorized microscopy stage, systematically sampling throughout the entire optic nerve section. In each section, an average of 22±2 sampling sites was manually counted using the CFI Plan Fluor 100× oil immersion objective. The manually counted axon counts ranged from 719 to 958 axons with a mean±S.D. of 826±72. The total number of axons in the optic nerve was estimated based on the stereological algorithm [30].

### Paraffin-embedded retinal sections

The retinas were embedded in paraffin and 5 µm sections were cut and collected on slides. Every 10^th^ slide was used for immunostainings. Briefly, retinal sections were deparaffinized and rehydrated, rinsed, pre-treated with 3% hydrogen peroxide for 10 min and incubated in 10% NGS in TBS for 30 min. Each primary antibody was incubated separately overnight at room temperature in a humidified chamber which was followed by washing in 0.05 M TBS, pH 7.4, several times for 5 min and incubation in secondary antibodies for 3 h at room temperature. Finally, the sections were rinsed in 0.05 M TBS, pH 7.4, and coverslipped. In the negative controls, the primary antibodies were omitted.

### *In situ* detection of DNA fragmentation

Paraffin embedded retinal sections were used for *in situ* detection of DNA fragmentation by terminal deoxynucleotidyl transferase-mediated dUTP nick end-labeling (TUNEL) according to the manufacturer’s instructions (ApopTag Peroxidase Kit S7101, Chemicon). Briefly, the retinal sections were deparaffinized in xylene and rehydrated in graded series of ethanol, pretreated with proteinase K (20 µg/ml, Sigma) for 15 min at room temperature and incubated in the equilibration buffer for 10 s. Then, working strength terminal deoxynucleotidyl transferase was applied in a humidified chamber for 1 h at 37°C. The sections were washed for 10 min in wash buffer and incubated in anti-digoxigenin-peroxidase for 30 min at room temperature. The reaction product was revealed using diaminobenzidine. Harris’s hematoxylin (Merck) was used as the counterstain.

### Statistical analysis

The data were analyzed using SPSS for Windows version 19.0 (SPSS Inc., Chicago, IL, USA) or GraphPad Prism version 5.00 for windows (San Diego, CA, USA). The groups were compared by t-test assuming equal variances. In cases where variances between the groups were significantly different (Levene's test), Mann-Whitney U-test was used. Friedman's ANOVA, followed by Dunn's post-hoc test, was used to detect age-dependent change in ERG in WT animals. The level of statistical significance was set at p<0.05. The values are presented as mean ± standard deviation (SD) if not stated otherwise.

## Results

### Electroretinography

**Figure 2:**
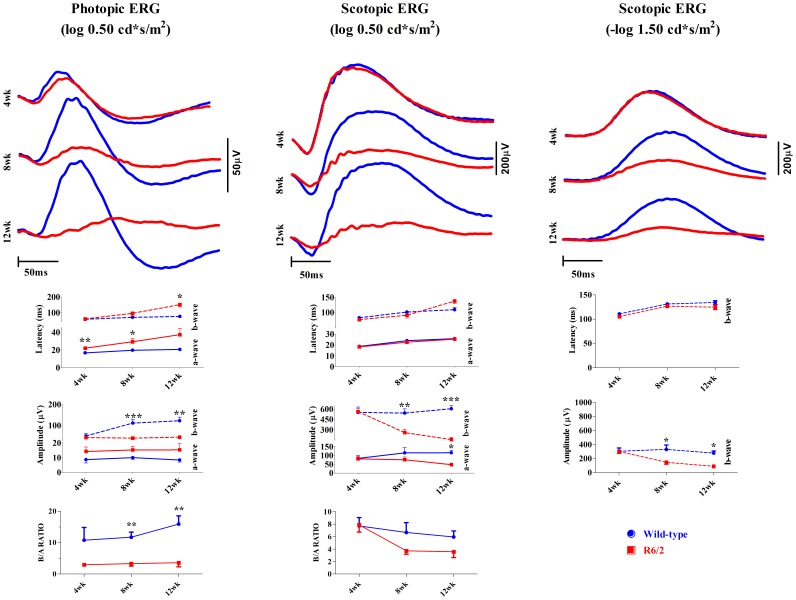
Progressively deteriorating ERG in R6/2 mice (in red) compared to their wild-type littermates (in blue). The uppermost rows represent the averaged ERG responses recorded at three different ages. Note that also wild-type responses change until animals reach a mature age. Solid lines represent a-wave statistics (mean±SEM) and dashed lines b-wave statistics in amplitude and latency graphs. B/a ratios presented on the lowest rows were calculated by dividing b-wave amplitudes by corresponding a-wave amplitudes. Statistically significant differences from t-test, or U-test in cases of unequal variances, are marked as *p<0.05; **p<0.01; ***p<0.001.

We used dark- and light-adapted flash and pattern stimulation to explore the functional integrity of specific retinal cells of R6/2 mice, and to get insight of their visual abilities. Already at 8 weeks of age, the retinal function was distorted in transgenic animals, although the within-group variability was high (Fig. 1 and 2).

#### Light-adapted state

The cone-pathway in R6/2 mice showed a clear change in response kinetics already at the age of 4 weeks (Fig. 2, left column). The latency of the a-wave was increased by almost 5 ms in R6/2 mice (mean±SEM: 22.0ms±1.05 [R6/2], 17.2 ms±0.90 [WT]; t = 3.11, df = 17, p<0.01). However, the response strength as measured by the a-wave (t = 1.67, df = 17, p = 0.11) or b-wave amplitudes (t = 0.84, df = 17, p = 0.41) did not differ between the groups nor did the b-wave latency (t = 0.64, df = 17, p = 0.53). By 8 weeks of age, the cone-mediated function in R6/2 mice was substantially impaired, and in two R6/2 mice the b-wave had completely disappeared (see Fig. 1C). The a-wave latency continued to increase in comparison to WT mice (mean±SEM: 29.3ms±3.62 [R6/2], 19.8ms±0.69 [WT]; z = −2.5, p<0.05) and also b-wave latency had a tendency to increase (paired t-test: t = 2,20, df = 7, p = 0.06). The b-wave amplitude at this age was much smaller in comparison to WT mice (z = 3.2, p<0.001), whereas the a-wave amplitude remained intact (see Fig. 2, left column). Between 8–12 weeks of age, the photopic ERG continued to deteriorate (Fig. 2, left column), which led to a significant increase also in the b-wave latency at 12-week group compared to WT mice (z = −2.7, p<0.05). The b-wave amplitude continued to decline compared to WT mice (z = −2.6, p<0.01). Overall, photopic ERG a- and b-wave latencies had a tendency to increase in R6/2 mice, whereas amplitudes did not change with age (Fig. 2, left column). However, within-subjects observations among R6/2 animals should be considered suggestive since the number of analyzed animals progressively decreased from 4 week (n = 12) to 8 week (n = 8) and 12 week (n = 3–4) age groups due to mortality and disqualification (absent b-wave). It should be noted that ERG response continued to increase in control WT animals as mice matured from 4 weeks to 12 weeks of age (see Fig. 2., left column; b-wave amplitude: x^2^ [3] = 8.9, p<0.01), which explains the progressively increasing gap in the b-wave amplitude between R6/2 and WT mice. The altered shape and the increased amplitude of the response from 4 weeks onwards may be explained by an increase in the eye size with accompanying increase in the pupil size [19] and possibly more optimal positioning of the ERG electrode. The b/a-wave amplitude remained similar over the course of the experiment within groups (see Fig. 2, left column lowest graph; WT: x^2^ [3] = 2.3, p = 0.4). The b/a-wave ratio was smaller in R6/2 mice already at 4 weeks (mean±SEM: 2.96±0.36 [R6/2], 10.8±4.05 [WT]; z = −1.9, p = 0.06) and the difference continued to increase at 8 weeks (mean±SEM: 3.31±1.65 [R6/2], 11.7±1.65 [WT]; z = −2.97, p<0.01) and further at 12 weeks of age (mean±SEM: 3.59±1.30 [R6/2], 15.9±2.64 [WT]; z = −2.97, p<0.01).

**Figure 3:**
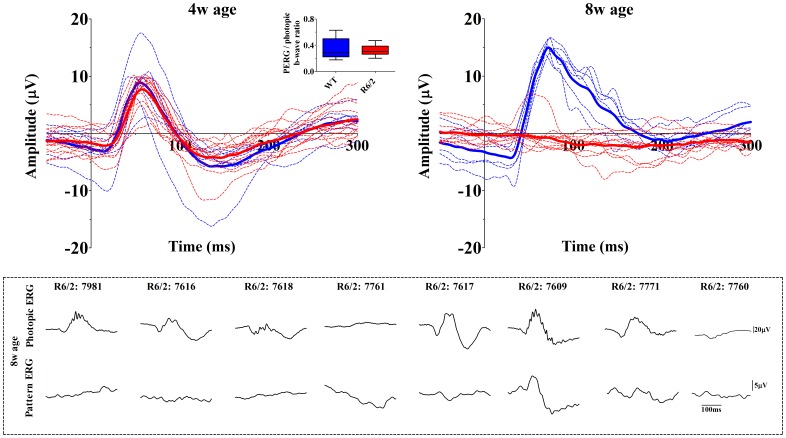
Pattern electroretinography (PERG). The larger graphs represent averaged (bolded) and invididual PERG traces (thin dashed lines) for R6/2 (blue) and wild-type (red) mice. The ratio analysis (insert) derived from PERG amplitude/photopic b-wave amplitude shows no difference between the groups at 4 weeks of age. However, at the age of 8 weeks most R6/2 mice have completely lost the PERG response (even though photopic ERG b-wave is still detectable), and thus no ratio analysis could be done. The dashed box illustrates individual photopic flash ERG and PERG responses for each R6/2 mice still having a remnant of photopic b-wave at the age of 8 weeks. Note that R6/2 mice 7981, 7616, 7617 and 7771 still have moderate photopic ERG responses but PERG is absent, and only mouse 7609 shows a detectable PERG response.

The RGC function at a light-adapted state (60 cd/m^2^) was assessed via the PERG responses. At 4 weeks of age, the groups did not differ significantly (PERG amplitude: z = −1.55, p = 0.13; b-wave latency: t = 0.89, df = 18, p = 0.39), although R6/2 mice had a slightly decreased amplitude (mean±SEM: R6/2 = 12.8±0.75 µV vs. WT = 15.9±2.21 µV; Fig. 3, left). However, even this small tendency disappeared when the ratio was calculated between PERG and photopic b-wave amplitudes (df = 17, t = 0.3842, p = 0.71) in order to isolate the specific RGC dysfunction instead of seeing downstream effect caused by dysfunction at first- or second-order neurons. At 8 weeks of age, nine out of ten R6/2 mice had lost their response to patterned stimuli, although four R6/2 mice lacking PERG still had moderate photopic ERG amplitude gain (b-wave amplitude mean±SEM: 45.10±14.7 µV [R6/2], 109.2±14.74 µV [WT]), normal b-wave latency (mean±SEM: 71.7±2.90 ms [R6/2], 70.37±1.78 ms [WT]) and normally appearing waveform (see Fig. 3 dashed box, animals 7981, 7616, 7617 and 7771). In fact, this subset of R6/2 mice exhibited an increase in photopic flash responses from 4 to 8 weeks of age as measured by b-wave amplitude (mean±SEM 33.5±6.26 µV [4w], mean±SEM 45.10±4.63 µV [8w]; df = 3, t = 3.86, p<0.05). Thus, one would expect to see corresponding PERG response in this subset of mice if RGC function was intact.

#### Dark-adapted state

At the age of 4 weeks, the R6/2 and WT groups did not differ with respect to the stimulus parameters assessing purely or primarily rod-driven pathways (weak flash stimulus, −1.5 log cd*s/m^2^; Fig. 2, right column). The pure rod-driven response in R6/2 mice remained normal in its waveform throughout the study, although it progressively weakened as compared to WT mice from the age of 8 weeks (b-wave amplitude: z = −2.5, p<0.01) to 12 weeks of age (z = −2.4, p<0.05). At 8 and 12 weeks of age, a stronger rod response (0.5 log cd*s/m^2^) showed a similar kind of progressive response degradation as cone-response although a- and b-wave latencies remained intact (Fig. 2, centermost graph). The a-wave amplitude was significantly decreased at 12 weeks of age (df = 8, t = 3.00, p<0.05) but not yet at 8 weeks (df = 13, t = 1.22, p = 0.24). The b-wave amplitude was significantly affected starting from 8 weeks (df = 13, t = 3.61, p<0.01) and progressing to 12 weeks of age (df = 8, t = 6.23, p<0.001). The more affected b-wave amplitude led to observable gap between b/a-wave ratios in R6/2 mice compared to WT mice (Fig. 2, center column), however, not reaching the statistical significance (8w: z = −1.27, p = 0.23; 12w: df = 1.43, t = 1.43, p = 0.19). Notably, the rod ERG was completely normal in R6/2 mice at 4 weeks of age, but decreased in amplitude substantially thereafter. These data indicate that the cone-pathway is primarily affected in R6/2 retinal degeneration, and that the rod-pathway dysfunction may be secondary.

### Histology

#### Number of RGCL neurons and astrocytes

**Figure 4:**
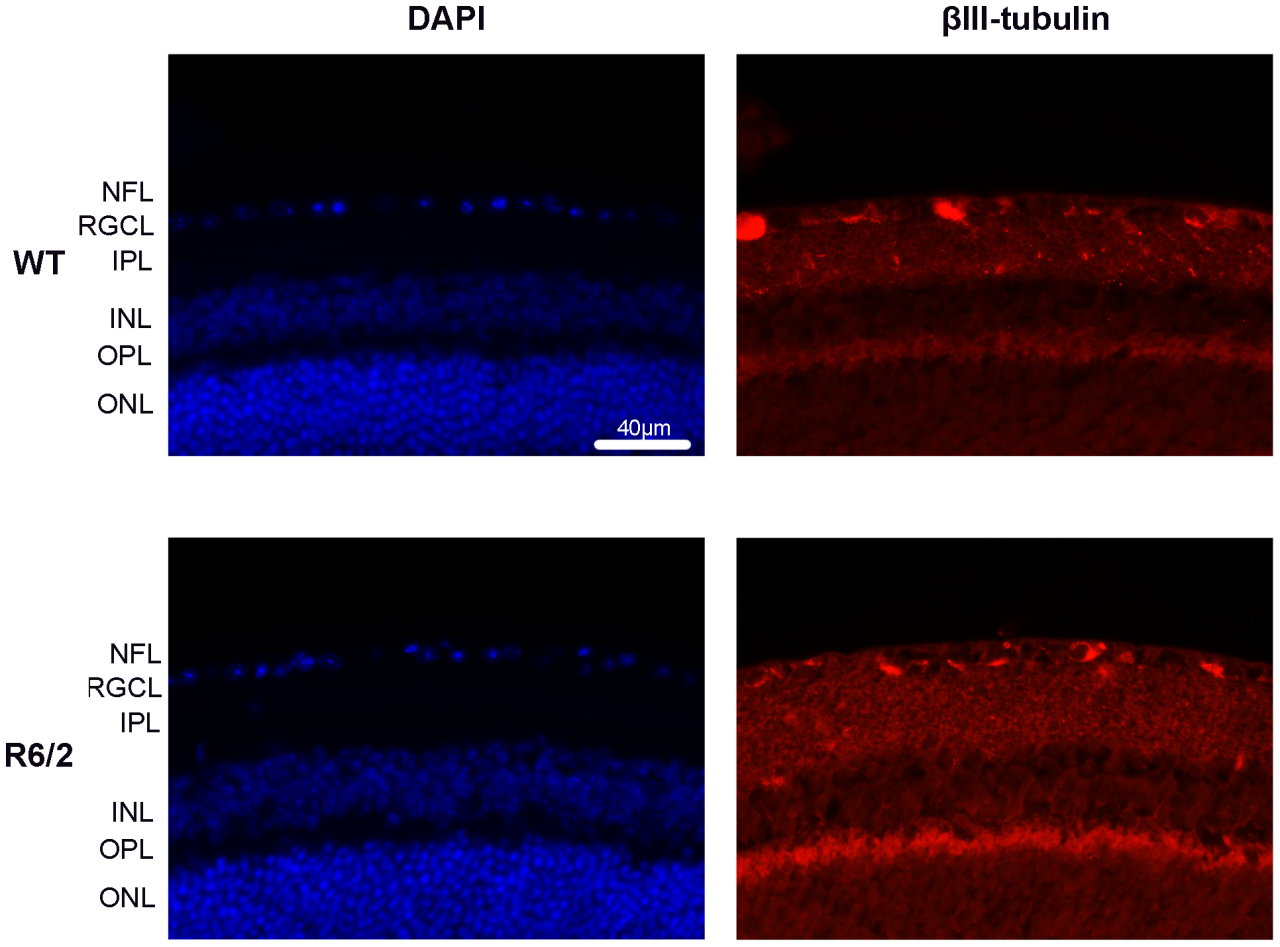
Images of β3-tubulin (in red) immunostained retinal sections from WT and R6/2 mice. The sections were counterstained for DAPI (in blue). Scale bar = 40 µm. NFL – nerve fiber layer, RGCL - retinal ganglion cell layer, IPL – inner plexiform layer, INL – inner nuclear layer, OPL – outer plexiform layer, ONL – outer nuclear layer.

The total number of DAPI-positive nuclei from RGCL was 100,628±17,818 and 103,300±13,527, and the total number of GFAP-ir cells from the NFL was 1,675±340 and 1,833±208 in the 18-week-old WT and R6/2 mice, respectively, in the retinal wholemounts. The number of β3-tubulin positive cells in the RGCL (Fig. 4) was 2.66±0.71/100 µm in the retinal sections of WT mice and 2.46±0.74/100 µm in the retinal sections of R6/2 mice, respectively. None of the markers displayed any statistically significant difference between the groups (Mann Whitney U-test, p≥0.28).

#### Optic nerve axon counts

**Figure 5:**
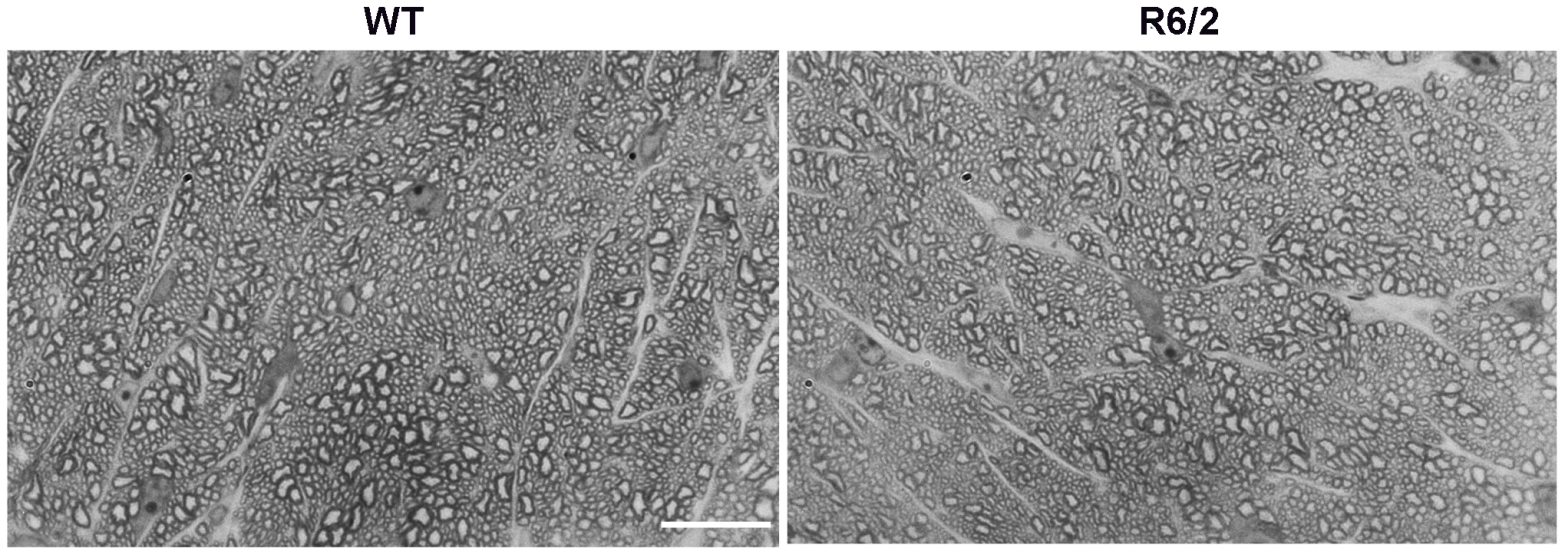
Representative images from semi-thin (1 µm-thick) optic nerve cross-sections of WT and R6/2 mice showing intact morphology of axons in 18-week old R6/2 mice. Scale bar = 15 µm.

Both the morphological appearance of optic nerves and their axons (Fig. 5) as well as the total number of axons did not differ between the groups of 18-week-old mice (41,543±4,523 in WT and 39,748±3,734 in R6/2 mice, Mann Whitney U-test, p = 0.54).

#### Accumulation of mHtt in retina and optic nerve. Retinal wholemounts

**Figure 6:**
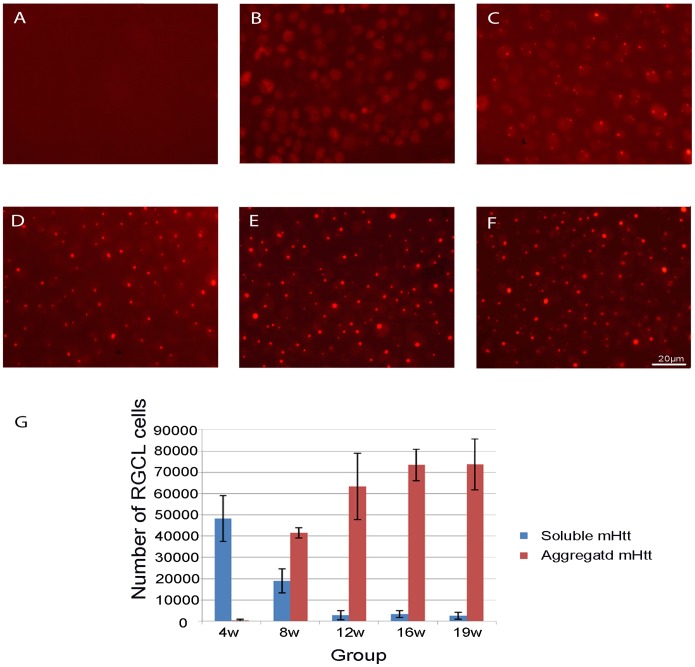
Images of mHtt deposits in RGCL in 4-week-old (B), 8-week-old (C), 12-week-old (D), 16-week-old (E) and 9-week-old (F) groups of R6/2 mice. Image A shows no corresponding mHtt staining in WT mouse retina. Soluble mHtt was predominant at the age of 4 weeks (B) in R6/2 mice, whereas at the age of 12 weeks (C) almost all RGCL cells contained aggregated form of mHtt. Scale bar = 20 µm. (G) Numbers of RGCL neurons having soluble vs. aggregated forms in R6/2 mice at different ages.

The mHtt staining in the retinal wholemounts of R6/2 mice was found at the age of 4 weeks with the majority of RGCL cells having a soluble mHtt immunostaining pattern and with only a few cells in the RGCL exhibiting mHtt-immunoreactive aggregates (Fig. 6B and G). No corresponding mHtt immunoreactivity was observed in WT mouse retinal wholemounts (Fig. 6A). At the age of 8 weeks, the number of RGCL cells that had mHtt positive aggregates in R6/2 mice significantly increased as compared to the 4-week-old R6/2 mice (Mann-Whitney U test, p<0.05; Fig. 6G). At a later age, the vast majority of RGCL cells contained the aggregated form of mHtt (Fig. 6D–F).

**Figure 7:**
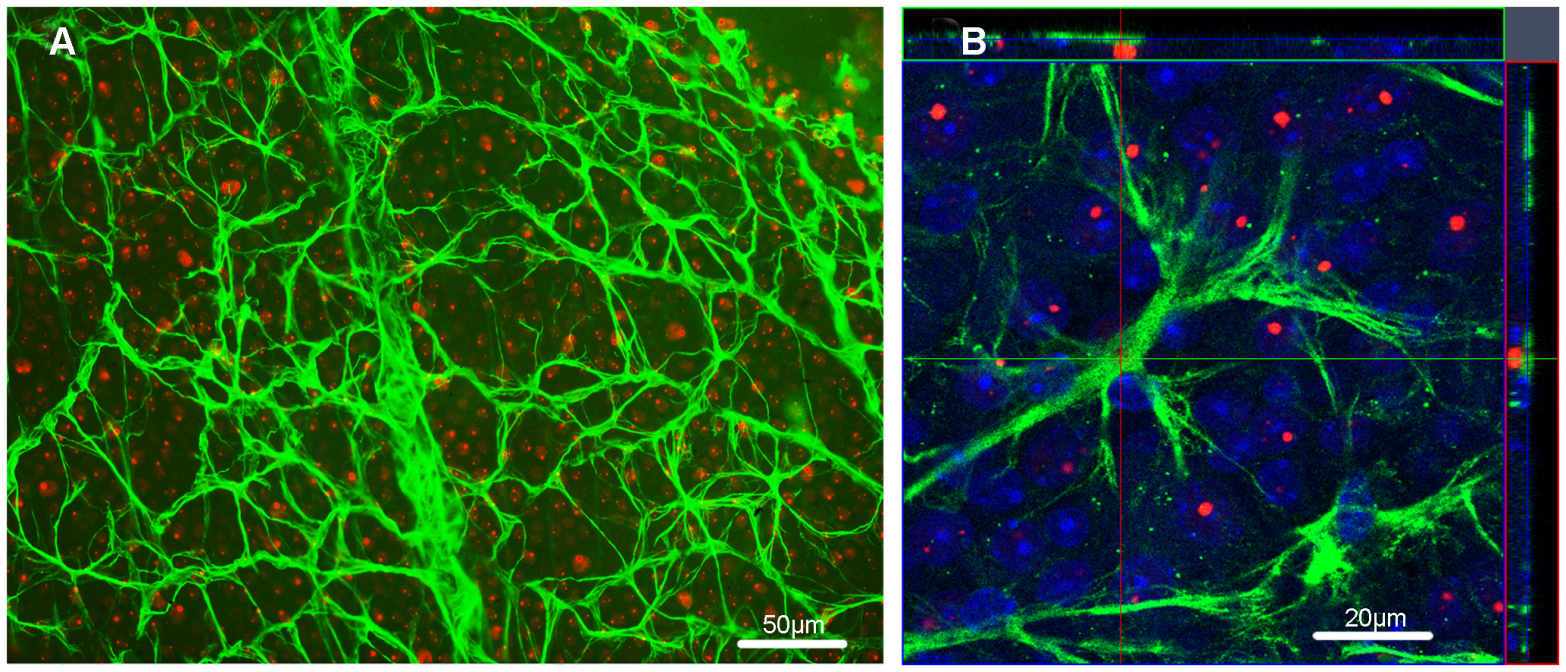
Colocalization of GFAP (green) and mHtt (red). (A) A low magnification picture illustrates GFAP-ir astrocytes and mHtt deposits from the retinal wholemount of 12-week-old R6/2 mouse. Scale bar = 50 µm. (B) A detailed confocal analysis of GFAP positivity, mHtt immunoreactivity and DAPI counterstain (blue) revealed no colocalization of GFAP and mHtt. Scale bar = 20 µm.

Next, we analyzed the wholemount retinas for mHtt deposition in GFAP-ir astrocytes (Fig. 7A). A few GFAP-ir astrocytes were detected, and they seemed to contain nuclear deposits of mHtt. However, a more detailed examination using the confocal analysis did not confirm GFAP and mHtt colocalization in the R6/2 mouse retinas (Fig. 7B).

#### Retinal sections

**Figure 8:**
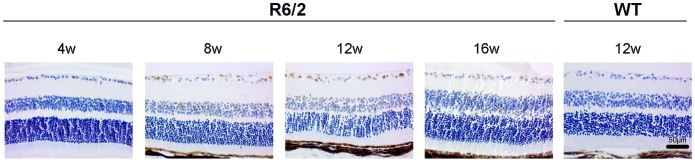
Deposits of mHtt in retinal sections of R6/2 mice in different age groups. Scale bar = 50 µm.

We used retinal sections to monitor mHtt deposition in different retinal layers while the HD-related pathology progresses in R6/2 mice. At all ages analyzed (4–18 weeks), the mHtt deposits were found in the RGCL, INL and some in ONL (Fig. 8). The number of mHtt-positive cells in R6/2 mice seemed to increase in the RGCL from 4- to 12-weeks of age with a slight decrease detected at 16-weeks of age. However, this is mainly an observational finding as we did not perform any quantitative measurements on the number of mHtt deposits from the retinal sections.

#### Optic nerve

**Figure 9:**
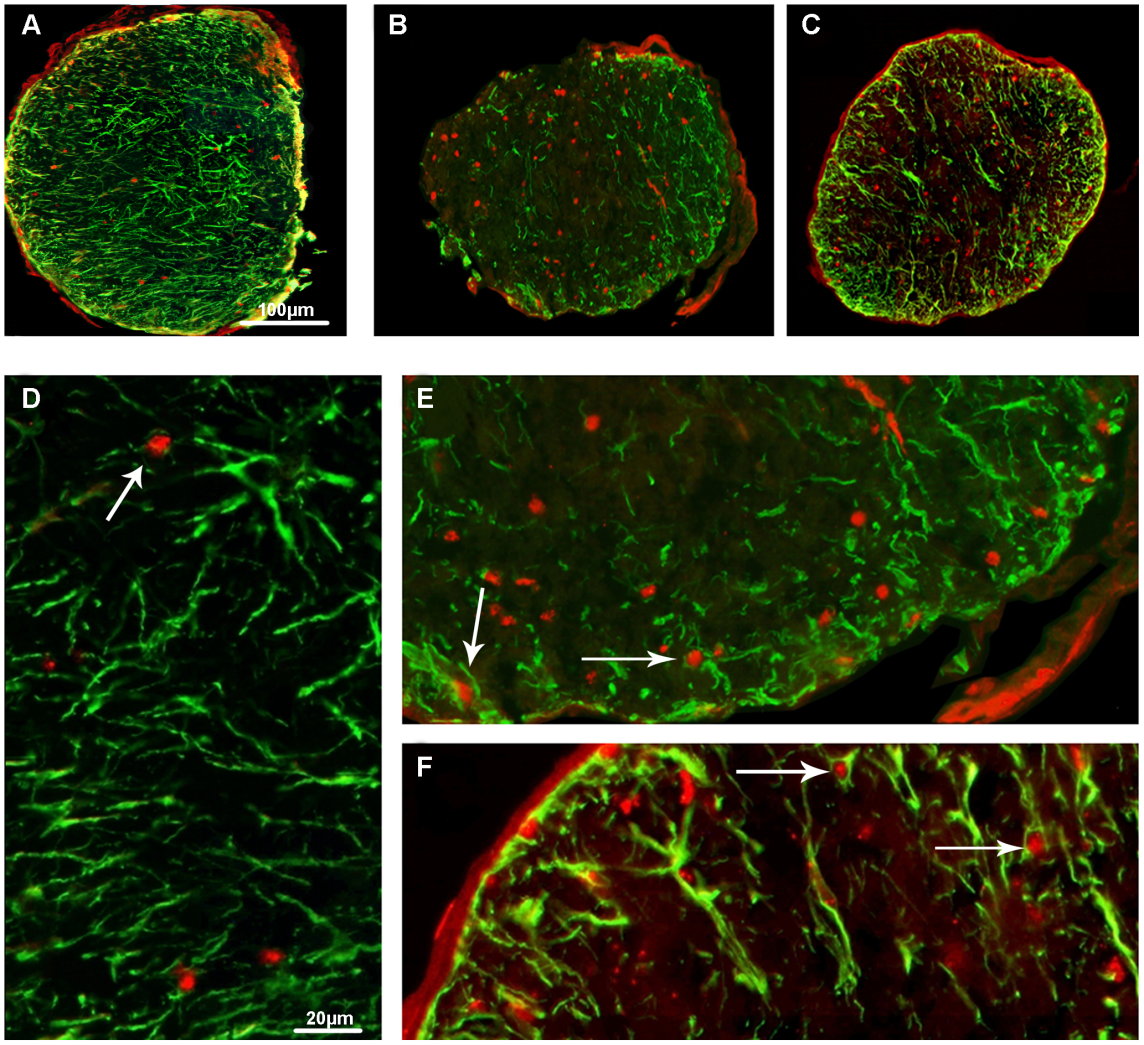
Colocalization of GFAP (green) and mHtt (red) in the optic nerve. (A–C) The number of mHtt immunoreactive deposits increased with age from 4-week-old (A) to 8-week-old (B) and 12-week-old (C) mice. Scale bar = 100 µm. (D–F) High magnification images of GFAP-immunoreactive astrocytes that colocalize mHtt in the optic nerve (indicated with arrows). Scale bar = 20 µm.

**Figure 10:**
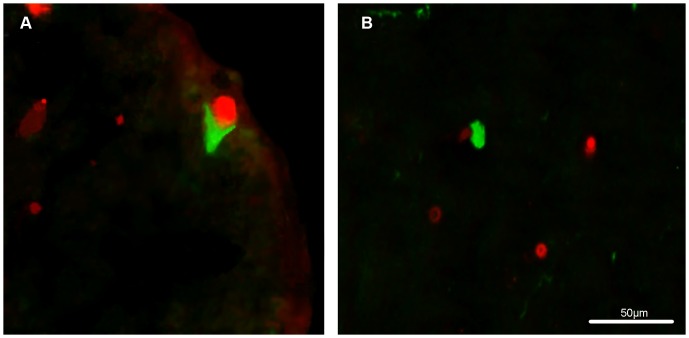
Some microglial cells (Iba-1 immunoreactivity in green) were found to engulf mHtt deposits (red) in the optic nerve of 12-week-old R6/2 mouse. Scale bar = 50 µm.

Similarly as with retinal sections, the mHtt immunoreactivity in the optic nerve of R6/2 mice showed an increasing pattern from ages of 4 weeks to 12 weeks (Fig. 9A and B, respectively). In contrast to the situation in the retina, mHtt was found in the GFAP-ir astrocytes of the optic nerve (Fig. 9D–F). Interestingly, we found Iba-1 positive microglia cells located close to the mHtt deposits in the optic nerve of R6/2 mice (Fig. 10).

#### Apoptosis (TUNEL) in the retina of R6/2 mice

**Figure 11:**
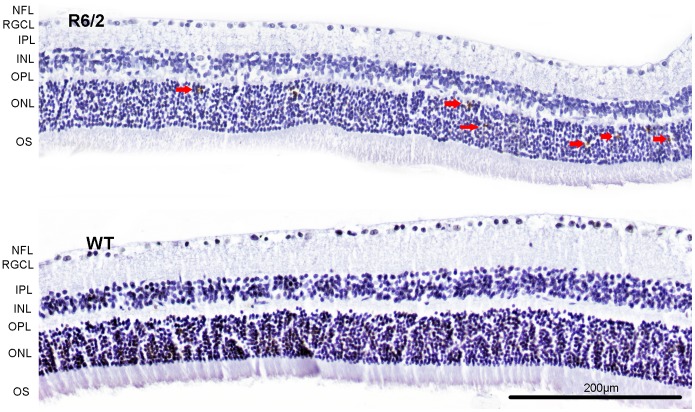
Apoptotic cells were found exclusively in the outer nuclear layer (ONL) of R6/2 mice as revealed by TUNEL staining (TUNEL positive cells are marked with red arrows). No corresponding staining was observed in WT mice. NFL – nerve fiber layer, RGCL - retinal ganglion cell layer, IPL – inner plexiform layer, INL – inner nuclear layer, OPL – outer plexiform layer, ONL – outer nuclear layer, OS – photoreceptor outer segments. Scale bar = 200 µm.

TUNEL-positive cells were found exclusively in the ONL in the R6/2 mice. Their number varied between 3–10 cells per section (Fig. 11) with no preference to any particular age group. No corresponding staining was observed in the WT mouse sections.

## Discussion

To the best of our knowledge, this is the first study that has followed RGC survival in detail through the development of progressive retinopathy in R6/2 mice. We characterized the PERG response in R6/2 mice at 4 and 8 weeks of age, observed the changes in the deposition of mHtt in RGCL cells from 4 to 18 weeks as well as estimated the number of cells and the number of axons in the optic nerve. In addition, we conducted an evaluation of the rod- and cone-mediated function in R6/2 mice starting as early as 4 weeks of age. Earlier studies in R6/2 mice have reported that the onset of degeneration in the outer retina occurs at the age of 10 weeks [14], [17], [31], and that there is a decline in photopic ERG starting from 6 weeks of age [14]. The present study suggests that impaired retinal function is present already at 4 weeks of age when soluble mHtt can be detected in retina. Even more importantly, the PERG was absent at 8 weeks of age in R6/2 mice. This should be taken into account in behavioral testing of R6/2 mice.

The retina is composed of layers of specialized neurons forming vertically (through the retina) and horizontally organized pathways. In the hierarchical vertical pathway, the photoreceptors (first-order neurons) sense and convert light into electrical, neuronal signals. These neuronal signals are passed to bipolar cells (second-order neurons) which finally feed these signals to optic nerve forming axons of RGCs (third-order neurons). While the functionality of photoreceptors and bipolar cells are readily obtainable by conventional ERG, the functional assessment of RGCs require more specific ERG techniques. Three distinct ERG methods are used to assess the functionality of RGCs: scotopic threshold response (STR; [32]–[34], photopic negative response [35], or pattern electroretinography (PERG, [36]). The PERG is recorded for contrast reversing pattern stimuli at a constant luminance thus providing a means to estimate retina’s ability to discriminate visual contours (see e.g. [20] for detailed description). Although PERG is a challenging method in mice, as it requires intact optics, it is well established (e.g. [20]–[21], [37]–[38]), and it has been shown to correlate with visual acuity [39]. Furthermore, the PERG is widely used in clinical practice [40]–[41], and it has been suggested to be the most sensitive measure of RGC dysfunction in mice [38]. It should be noted, however, that all above-mentioned functional assessments of RGCs are somewhat compromised if dysfunction occur already before RGCs at the retinal hierarchy.

We assessed inner retinal function and pattern discrimination ability by measuring PERG. The PERG was similar between WT and R6/2 mice at the age of 4 weeks, but by 8 weeks of age it was practically abolished in the R6/2 mice. Since outer retinal function, as assessed with photopic ERG, was also substantially decreased by this age, the cone-driven PERG signal disappearance can be mostly explained by diminished input from cone bipolar cells. However, a subset of R6/2 mice still had comparable photopic ERG responses between 4 and 8 weeks of age, but yet completely lacked PERG at 8 weeks of age (see Fig. 3, dashed box). All mice had representative PERG responses at 4 weeks of age (Fig. 3., top left). Would RGC processing be intact, the PERG response should remain if photopic ERG does not significantly decrease. Although PERG recording in mice is a relatively new method, and thus gain relationships between photopic ERG and PERG is yet to be determined, it is likely that cone bipolar cells monotonically drive PERG response in mesopic and moderate light levels in healthy retina. For example, Porciatti and colleagues [42] showed that the mouse pattern evoked visual potentials (VEPs), which have a counterpart in PERG [39], almost linearly decreased in amplitude as luminance was stepwise decreased from 25 cd/m^2^ to 0.25 cd/m^2^, reaching noise level at the lowest luminance. They concluded that pattern VEPs are primarily cone-driven in C57BL/6J mice. In primates, the PERG has been shown to be luminance dependent as well [43]. Thus, although we could indisputedly show dysfunction only upstream to RGCs (see discussion below), the PERG and photopic ERG comparison at 8 weeks of age imply dysfunction also at RGCs.

A recent study by Li and colleagues [14] found that scotopic (rod-mediated) ERG responses did not differ significantly between R6/2 and WT mice until the age of 9 weeks, whereas photopic ERG amplitudes (cone-mediated responses) in R6/2 mice were significantly lower than those in WT mice already at their first measurement conducted at 6 weeks of age. Our FERG data provide evidence that cone-mediated function is affected in R6/2 mice already at the age of 4 weeks. In normal photopic ERG, the negative a-wave, which is caused by the photoreceptor activation [44], is rapidly overwhelmed by a positive going b-wave since the photoreceptor activation causes ON-bipolar cells to be depolarized via glutamatergic excitation [45]. In the 4-week-old R6/2 mice, this polarity shift (a-wave through) came approximately 5 ms later than in WT mice. In addition, even at the age of 8 weeks, when the photopic ERG waveform had drastically diminished in R6/2 compared to WT mice, the a-wave appeared to be similar in amplitude in both genotypes (Fig. 2, left column), whereas the b-wave was dramatically diminished. Over the time course of the ERG follow-up, the photopic b/a-wave amplitude ratio was smaller in R6/2 mice. Taken together, the early and strong latency shift and small b/a-wave amplitude ratio in R6/2 mice indicate that signal transmission from cones to cone bipolar cells is the first and most pronounced functional deficit in R6/2 retinas.

Although occurring substantially later in onset and less drastic as compared to the cone-pathway, the rod-pathway also showed functional deficit starting from 8 weeks of age in R6/2 mice, which is slightly earlier than that reported by Li et al. [14]. The fractional discrepancy between the studies is likely caused by different experimental designs. As the rod-pathway remains functional longer than the cone-pathway in the R6/2 mouse, and rod-ERG with preserved waveforms (no overt latency shifts, see Fig. 2) provides another opportunity to study RGC specific dysfunction *in vivo* by STR. Although STRs would be inevitably also affected by upcoming outer retina defects in R6/2 mice, Nguyen and colleagues [33] recently established a sophisticated computational rod-ERG gain analysis method to distinguish specific inner retina defects from downstream effects caused by outer retinal dysfunction. Thus, future *in vivo* work could be directed to focus on early changes at rod-pathway. However, one should note that there is a notable limitation for longitudal ERG studies in this mouse line. Anesthesia required for ERG should be kept short, and thus ERG protocol should be efficient as these animals get very diseased with age and thus do not easily recover from anesthesia. We tackled this issue by using minimal, optimized anesthetic dose, recording only few parameters and using simple ERG technique which led to short anesthesia time. Anesthesia was reversed by α2-antagonist, atipamezole. In addition, we recorded electrocardiogram during the anesthesia to follow vital functions. In practical terms, ERG signals are strong predictors of visual functions (especially the PERG) and our ERG results indicate that the vision of R6/2 mice is poor already at a young age and they may be unable to discriminate visual patterns already by 8 weeks of age. Night vision may also be eventually severely affected.

Although the nuclear mHtt is more predominant in neurons than in glial cells [46]–[47], it is still unclear whether mHtt expression in glial cells contributes to HD-related pathology. However, there is recent evidence indicating that disturbed glutamate uptake by astrocytes may lead to glutamate excitotoxicity of the medium spiny striatal neurons in HD. In transgenic mice that selectively express N-terminal mHtt in astrocytes, the presence of mHtt in astrocytes decreased the expression of glutamate transporter and reduced glutamate uptake [48]. In our study, retinal astrocytes were devoid of mHtt, whereas mHtt-immunoreactive aggregates were found in the astrocytes of the optic nerve of the R6/2 mice. However, the number of optic nerve axons and their morphological appearance indirectly indicated that the presence of mHtt in optic nerve astrocytes had not affected the optic nerve milieu.

## Conclusions

Here we describe early functional deficits in R6/2 mice starting from the age of 4 weeks which led to disappearance of PERG signal by 8 weeks of age. No observable pathology or degeneration of RGCs or their axons was apparent in the optic nerve up to 19 weeks of age. This kind of early deterioration in visual function in R6/2 mice may provide a new means to monitor responses to treatments counteracting the effects of mHtt and should be taken into account in behavioral testing.
